# Supplementary material for: Structures in G proteins important for subtype selective receptor binding and subsequent activation
Source: Commun Biol. 2021 May 27;4:635. doi: 10.1038/s42003-021-02143-9 (PMC8160216; doi:10.1038/s42003-021-02143-9)
Supplement: Supplementary file 2 — Supplementary Information [file 42003_2021_2143_MOESM2_ESM.pdf]

# Supplementary Information

# Structures in G proteins important for subtype selective receptor binding and subsequent activation

**Volker Jelinek[1], Nadja Mösslein[1], Moritz Bünemann[1]**

[1]Institute of Pharmacology and Clinical Pharmacy, Philipps-University Marburg, Marburg, Germany

## Supplementary Figures

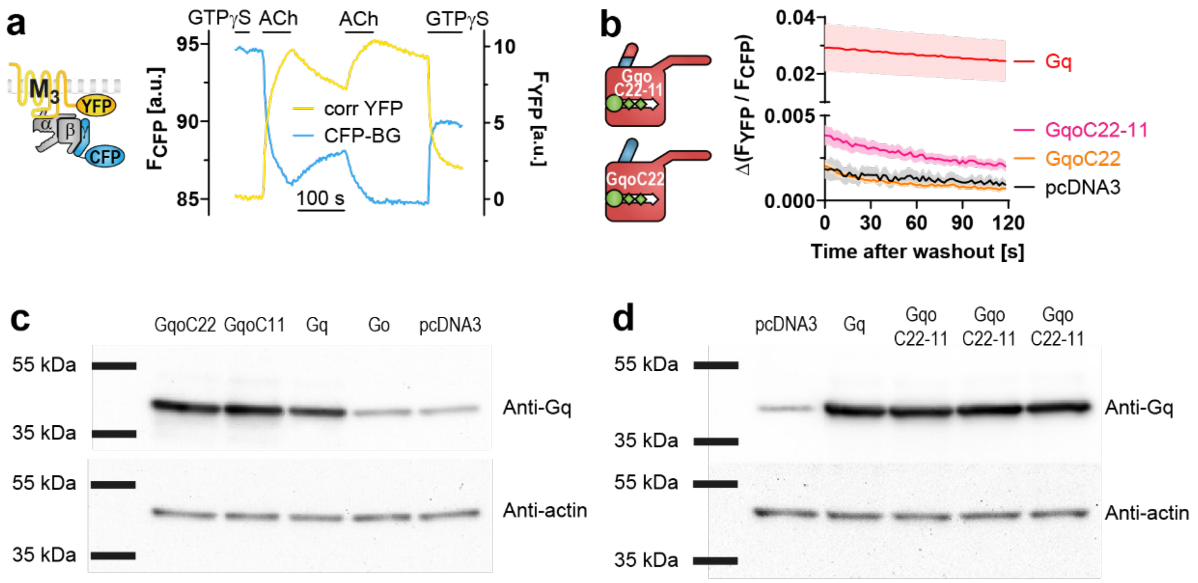


**Supplementary Fig. 1 Binding of G proteins to $M_3$ receptors in permeabilized cells.** G protein binding experiments in permeabilized HEK293T cells were performed in analogy to **Fig. 1b**. **a** Individual emissions of YFP and CFP are shown during a measurement investigating Gq binding to $M_3R$. YFP emission was corrected for the background fluorescence, the spillover of CFP into the YFP channel and the direct YFP excitation at 420-450 nm (corr-YFP). CFP emission was corrected for the background fluorescence (CFP-BG). **b** The schemes of GqoC22-11 and GqoC22 depict the exchanges of different parts of the $\alpha5$ helix in $G\alpha q$ by $G\alpha o$. Averaged traces of absolute amplitudes for Gq (red, n=11), GqoC22-11 (pink, n=11), GqoC22 (orange, n=14) and pcDNA3 (black, n=12 duplicated from **Fig. 1b**) binding to $M_3R$ illustrate that no clear FRET signals of GqoC22-11 and GqoC22 over endogenous G proteins (pcDNA3 as empty vector transfected instead of $G\alpha$) were detectable. Data points are represented as means $\pm$ SEM from three independent experiments. **c** A representative immunoblot (out of three independently performed blots) of cells transfected with mCitrine-labeled $M_3$ receptors, the respective $G\alpha$ subunit, $G\beta_1$ and mTurquoise2-labeled $G\gamma_2$ subunits illustrates similar expression levels of GqoC22 compared to GqoC11, Gq and empty vector. Much weaker bands were observed in cells transfected with Go and pcDNA3, most likely reflecting endogenously expressed $G\alpha q$ subunits so that the specificity of the anti-Gq antibody towards Gq-based constructs was verified. **d** The second immunoblot performed in analogy to **c** shows equal expression of GqoC22-11 (three independent transfections) and Gq.

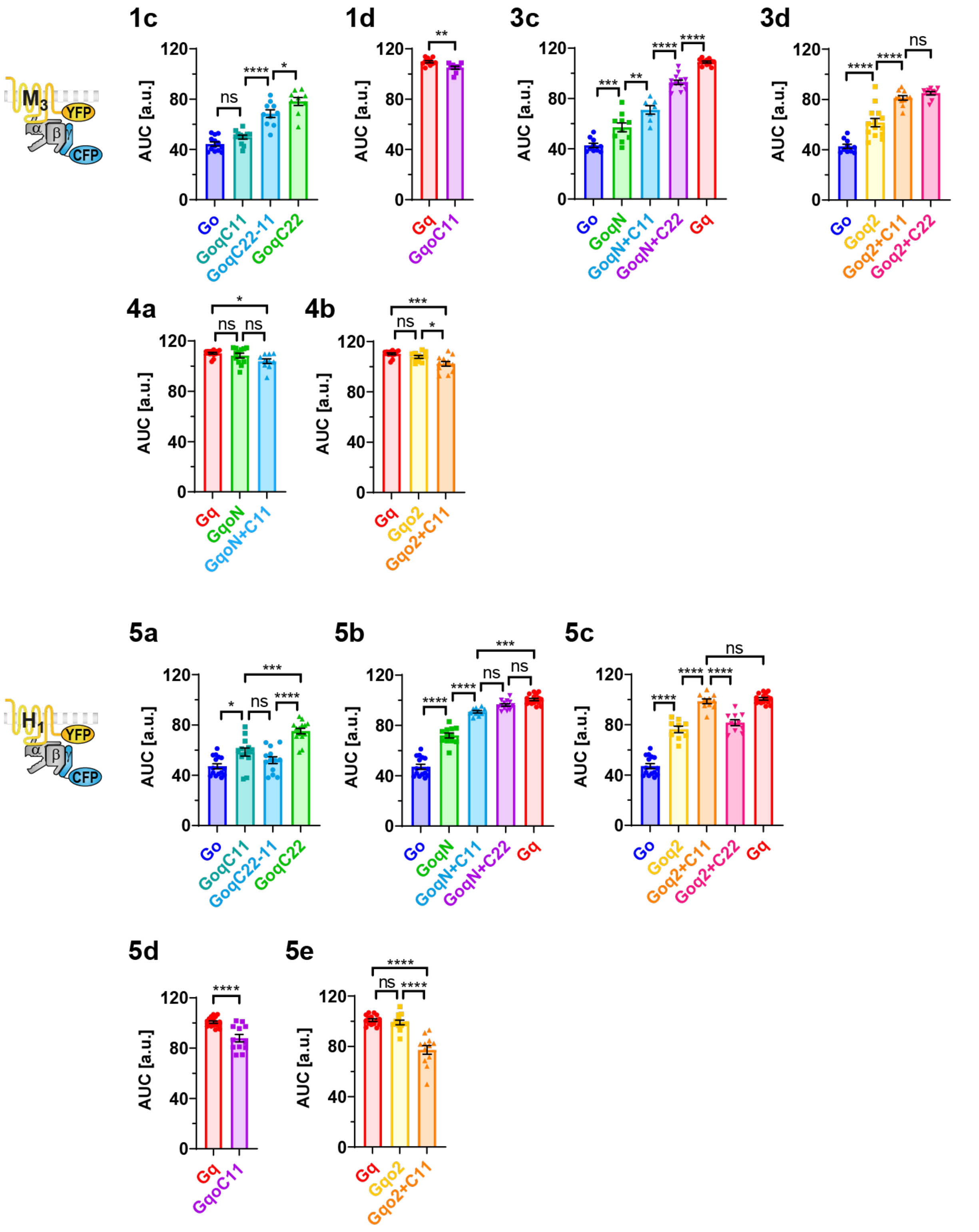


**Supplementary Fig. 2 Summary of absolute AUC values.** Absolute AUC values represent the complex stability of G proteins bound to $M_3R$ (first row) or to $H_1R$ (second row) in nucleotide depleted cells as described in **Fig. 1b**. Each graph is named after the Fig. to which it is related. All data points are represented as means ± SEM. The number of cells (n's) and statistical analyses are described in the related Figures.

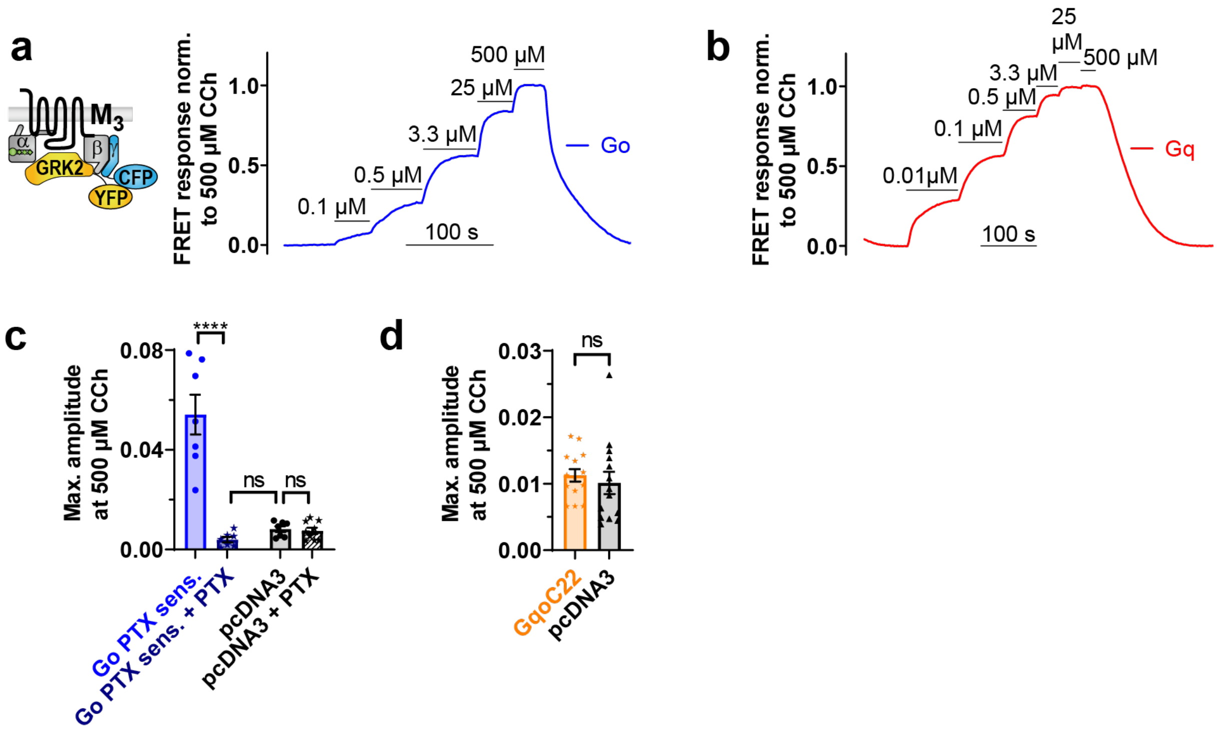


**Supplementary Fig. 3 Indirect activation of G proteins by M₃ receptors.** Indirect G protein activation measurements with M₃R in intact HEK293T cells were performed in analogy to **Fig. 2a,b**. Representative cells are shown of Go (**a**) and Gq (**b**) normalized to the maximum concentration of 500 µM CCh. **c** Endogenous G protein activation was measured in response to a pertussis toxin (PTX) pretreatment overnight with a PTX concentration of 50 ng $mL^{-1}$. As a positive control, responses of transfected PTX sensitive Go subunits (lighter blue, Go PTX sens.; n=7) are shown to be reduced by a PTX pretreatment (dark blue, Go PTX sens. + PTX; n=7) to the level of pcDNA3 (black, pcDNA3; n=7). As endogenous signals (pcDNA3) could not be further reduced by PTX (black striped, pcDNA3 + PTX; n=9) they presumably did not originate from the Gi/o family. Data points are represented as means ± SEM from two independent experiments. **d** Maximum amplitudes of GqoC22 (orange, n=14) and pcDNA3 (black, n=14) showed no specific signals of GqoC22 over endogenous proteins even though proper expression of GqoC22 was confirmed by immunoblotting (**Supplementary Fig. 1c**). Data points are represented as means ± SEM from three independent experiments. Statistical analyses were performed using a one-way ANOVA followed by Tukey's posttest (**c**, ****$P < 0.0001$, ns if $P \geq 0.05$) or a one-tailed t-test (**d**, ns if $P \geq 0.05$).

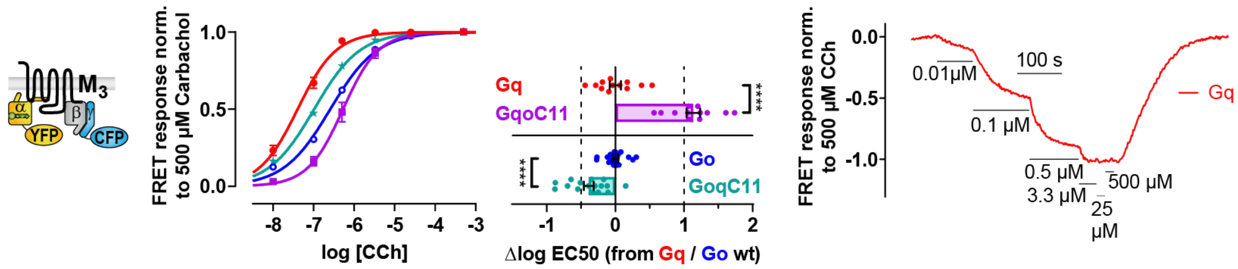


**Supplementary Fig. 4 Direct activation of G proteins by M₃ receptors.** Direct G protein activation measurements with the M₃R were performed in intact HEK293T cells in analogy to the indirect activation measurements (**Fig. 2a,b**). In contrast, FRET decreases could directly be measured upon dissociation of activated Gα from Gβγ subunits with cells transfected with M₃R, various YFP-labeled Gα subunits, $G\beta_1$ and N-terminally mTurquoise2-labeled $G\gamma_2$ subunits. A representative trace is shown in the right graph. All other data points are represented as means ± SEM from three independent experiments and are colored as indicated in the bar graph (Gq; n=12, GqoC11; n=12, Go; n=17 and GoqC11; n=16). Statistical analyses were performed using two one-tailed t-tests (****P < 0.0001).

**a**

| Goq names | Gqo names | Number of amino acids | Structure | Amino acids | Position (CGN) |
|---|---|---|---|---|---|
| GoqN | GqoN | 34 / 40 | αN - β1 | Go: M1-V34<br>Gq: M1-L40 | G.HN.01 -<br>G.S1.02 |
| Goq1 | Gqo1 | 11 | αN - β1 | Go: K24-V34<br>Gq: R30-L40 | G.HN.48 -<br>G.S1.02 |
| Goq2 | Gqo2 | 6 | β2 - β3 | Go: T191-H196<br>Gq: D195-I200 | G.S2.07 -<br>G.S3.02 |
| Goq4 | Gqo4 | 9 | α4 - β6 | Go: K311-I319<br>Gq: L316-I324 | G.H4S6.01 -<br>G.S6.01 |
| GoqC11 | GqoC11 | 11 | α5 | Go: I344-Y354<br>Gq: L349-V359 | G.H5.16 -<br>G.H5.26 |
| GoqC22 | GqoC22 | 22 | α5 | Go: Q333-Y354<br>Gq: R338-V359 | G.H5.05 -<br>G.H5.26 |

**b**

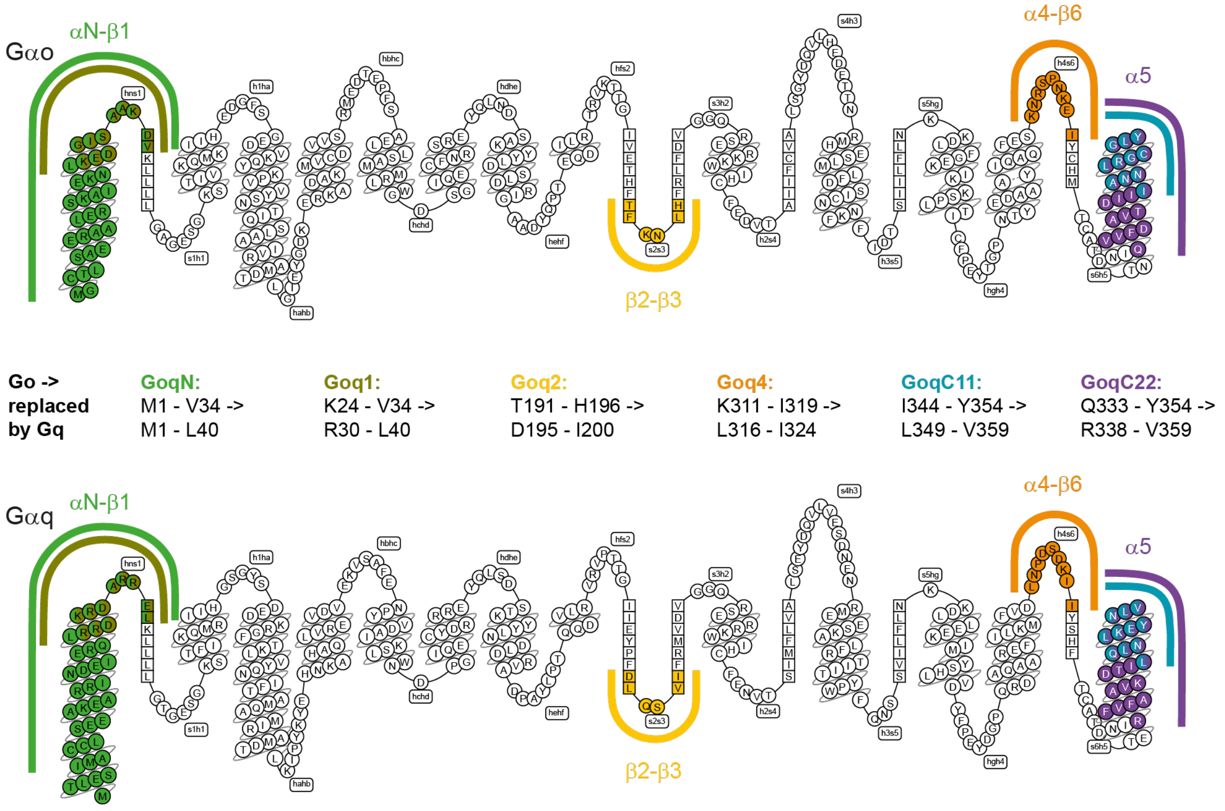


**Supplementary Fig. 5 Detailed overview of Gα chimeras. a** Positions and amount of amino acids swapped in Gα chimeras are listed. **b** Snake plots (obtained from gpcrdb.org) illustrate the amino acid sequences of Gαo and Gαq which are color-coded for chimeric exchanges. Colors are related to the respective Gα chimeras that are indicated in the middle.

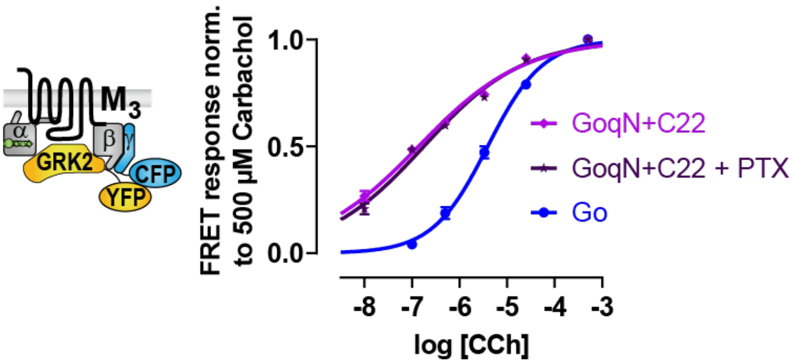


**Supplementary Fig. 6 Influence of pertussis toxin (PTX) on indirect G protein activation measurements.** Experiments were performed in analogy to **Fig. 2a,b** with GoqN+C22 (itself PTX insensitive because of the C-terminus of Gαq) activated by $M_3R$. The concentration-response curve of GoqN+C22 + PTX was measured upon a PTX pretreatment overnight with a PTX concentration of 50 ng $mL^{-1}$. The slope could not be steepened suggesting that the flattening of the curve could not be attributed to the interference of endogenous Gi/o proteins. A positive control showing proper PTX function is provided in **Supplementary Fig. 3c**. All data points are represented as means ± SEM from two independent experiments (GoqN+C22; n=8, GoqN+C22 + PTX; n=8, and Go; n=8).

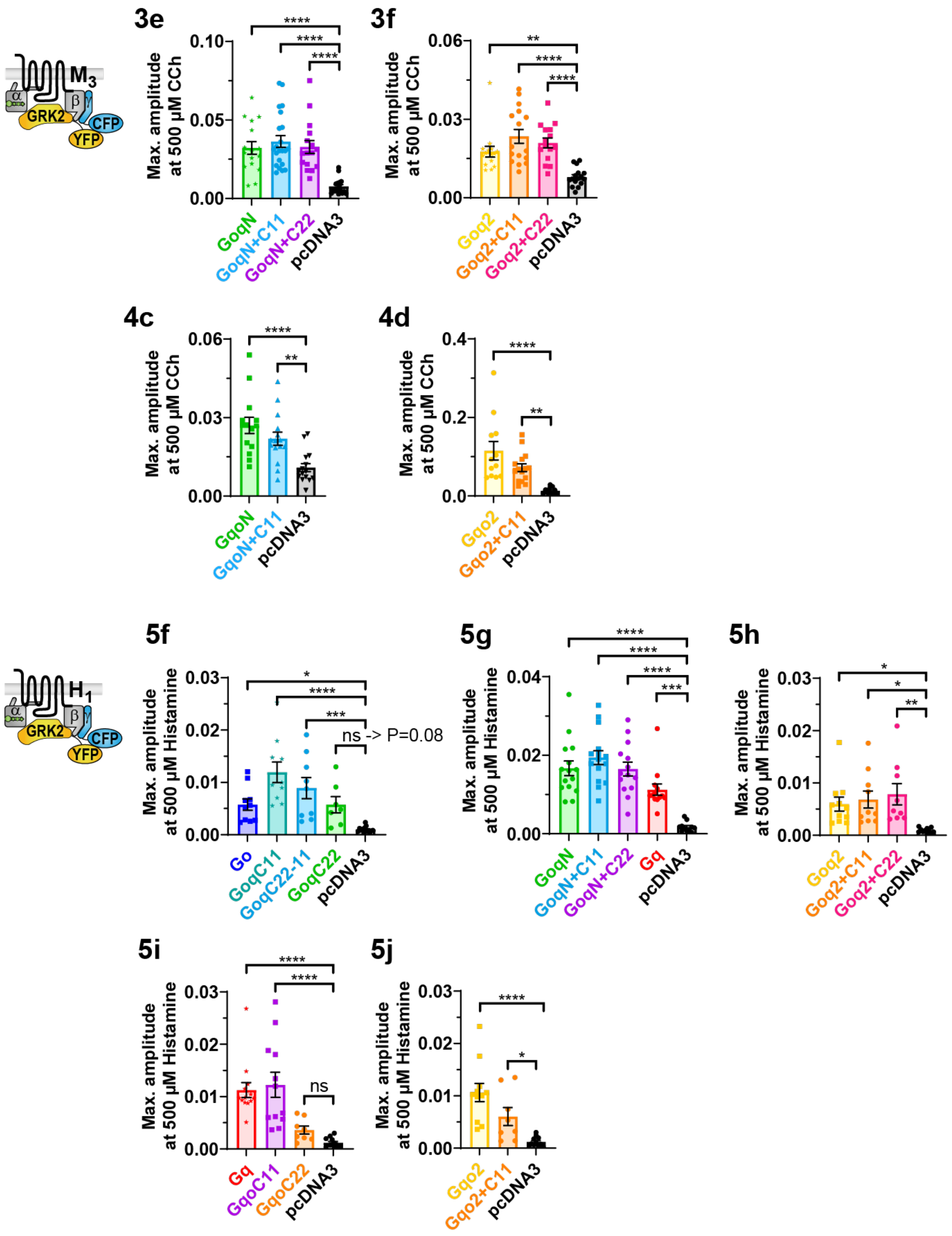


**Supplementary Fig. 7 Summary of maximum amplitudes.** Maximum amplitudes of indirect G protein activation measurements performed in analogy to **Fig. 2a,b** are depicted for $M_3R$ (first row) and $H_1R$ (second row) if not included elsewhere. Specific signals verified by significantly higher amplitudes of chimeric G$\alpha$ subunits at the maximum agonist concentration compared to cells transfected with empty vectors (pcDNA3) instead of G$\alpha$ subunits. Each graph is named after the Fig. to which it is related. All data points are represented as means ± SEM. The number of cells (n's) and statistical analyses are described in the related Figures.

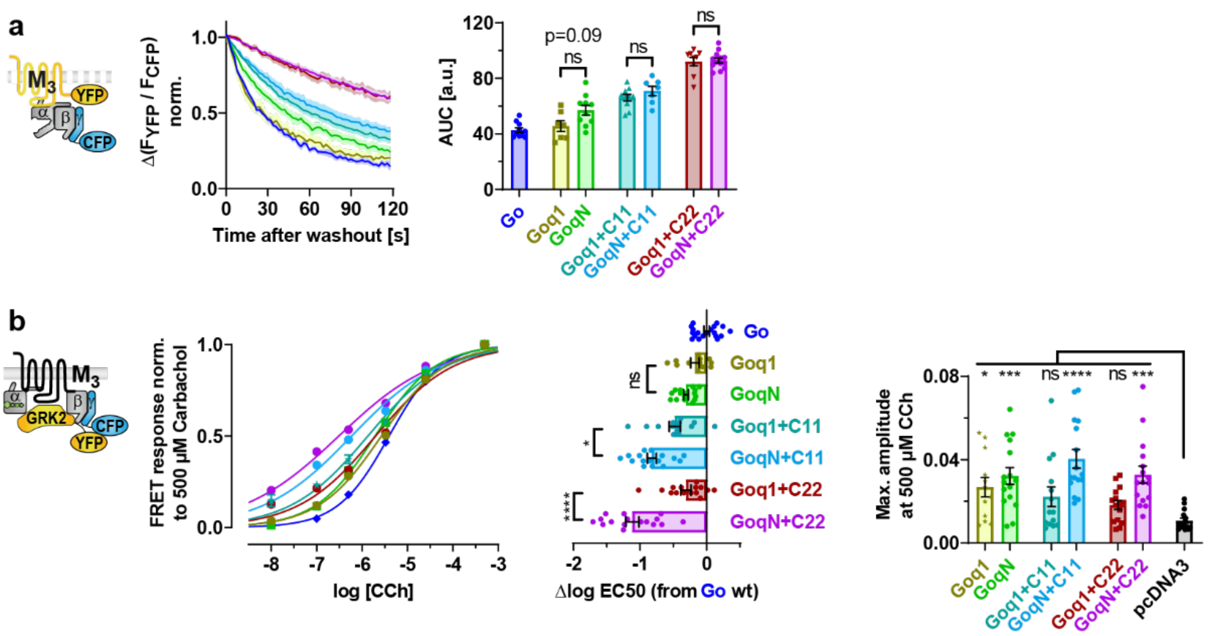


**Supplementary Fig. 8 Comparison of binding and activation measurements of Goq1 and GoqN chimeras with the M₃ receptor. a** G protein binding experiments in nucleotide-depleted HEK293T cells were performed in analogy to **Fig. 1b**. Dissociation kinetics of Goq1 constructs from M₃R were mostly similar to the GoqN counterparts. (Go; n=10, Goq1; n=7, GoqN; n=10, Goq1+C11; n=10, GoqN+C11; n=7, Goq1+C22; n=9 and GoqN+C22; n=13). **b** Indirect G protein activation measurements with M₃R in intact HEK293T cells were performed in analogy to **Fig. 2a,b**. Small amplitudes for Goq1+C11 or Goq1+C22 revealed no specific activation over endogenous Gα subunits (pcDNA3) and therefore they exhibited hardly any potency shifts. (Go; n=19, Goq1; n=12, GoqN; n=16, Goq1+C11; n=14, GoqN+C11; n=16, Goq1+C22; n=15, GoqN+C22; n=16 and pcDNA3; n=15). All data points are represented as means ± SEM from at least three independent experiments and are colored as indicated in the bar graphs. Statistical analyses were performed using one-way ANOVAs followed by Tukey's posttests for comparison of AUC and EC50 values or Dunnett's posttest to compare against pcDNA3 for the maximum amplitudes (**a-b**, *P < 0.05, ***P < 0.001, ****P < 0.0001, ns if P ≥ 0.05).

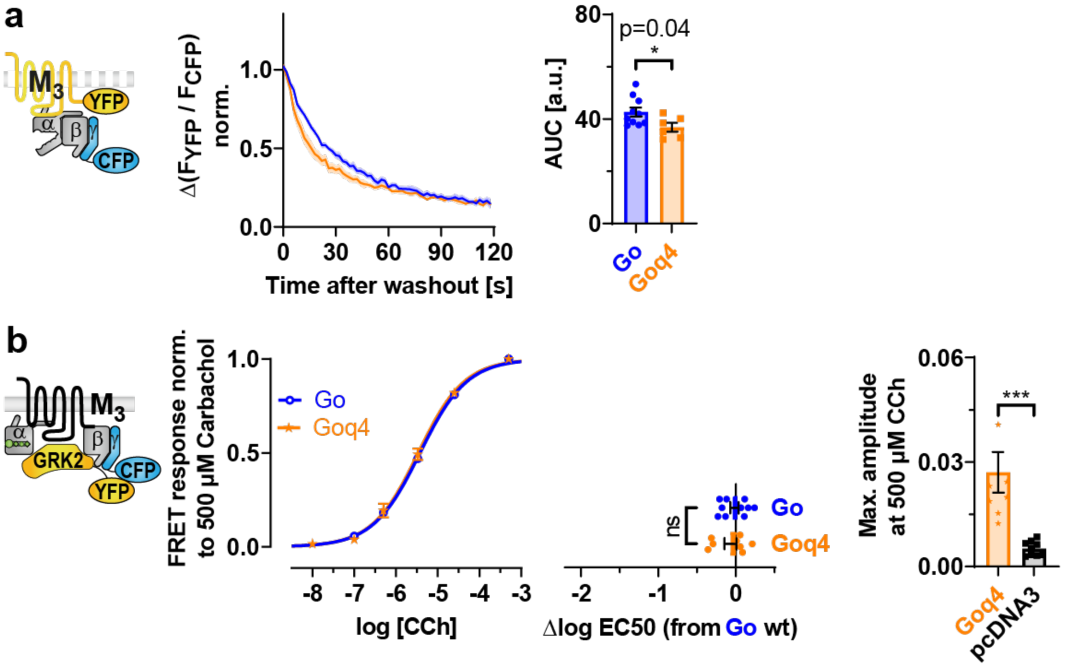


**Supplementary Fig. 9 Binding and activation measurements of Goq4 chimeras with the M$_3$ receptor.** Goq4 chimeras containing the α4/β6 loop of Gαq were investigated with the M$_3$R. **a** G protein binding experiments were performed in analogy to **Fig. 1b**. The averaged trace of Goq4 (orange; n=6) ran early to the same plateau as Go (blue; n=10) and was therefore barely different. **b** Indirect G protein activation measurements were performed in analogy to **Fig. 2a,b**. There was no detectable difference between Goq4 (orange) and Go (blue). (Go; n=10, Goq4; n=8; pcDNA3; n=9). All data points are represented as means ± SEM from at least two independent experiments. Statistical analyses were performed using two-tailed t-tests for comparison of AUC and EC50 values or a one-tailed t-test to compare against pcDNA3 for the maximum amplitudes (**a-b**, ***P < 0.001, *P < 0.05, ns if P ≥ 0.05).

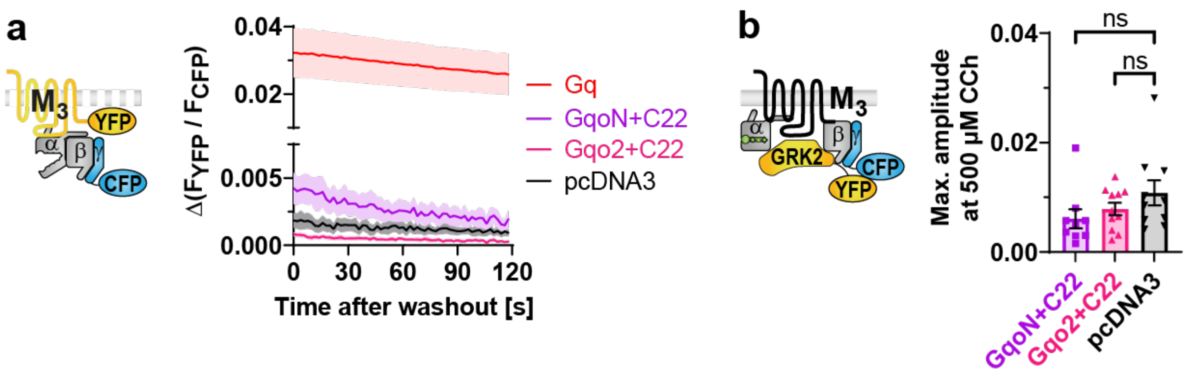


**Supplementary Fig. 10 GqoC22 double chimeras that cannot be bound or activated by the M$_3$ receptor. a** G protein binding experiments were performed in analogy to **Fig. 1b**. Averaged traces of absolute amplitudes for Gq (red; n=12), GqoN+C22 (purple; n=8), Gqo2+C22 (pink; n=10) and pcDNA3 (black; n=12) binding to M$_3$R illustrate that there was no detectable signal of GqoN+C22 and Gqo2+C22 over endogenous G proteins (pcDNA3 as empty vector transfected instead of G$\alpha$). **b** Indirect G protein activation measurements were performed in analogy to **Fig. 2a,b**. Maximum amplitudes of GqoN+C22 (purple; n=9), Gqo2+C22 (pink; n=11) showed no differences compared to activation of endogenous proteins (pcDNA3, black; n=10). All data points are represented as means ± SEM obtained from at least two independent experiments. Statistical analyses were performed using a one-way ANOVA followed by Dunnett's posttest to compare against pcDNA3 for the maximum amplitudes (ns if P ≥ 0.05).

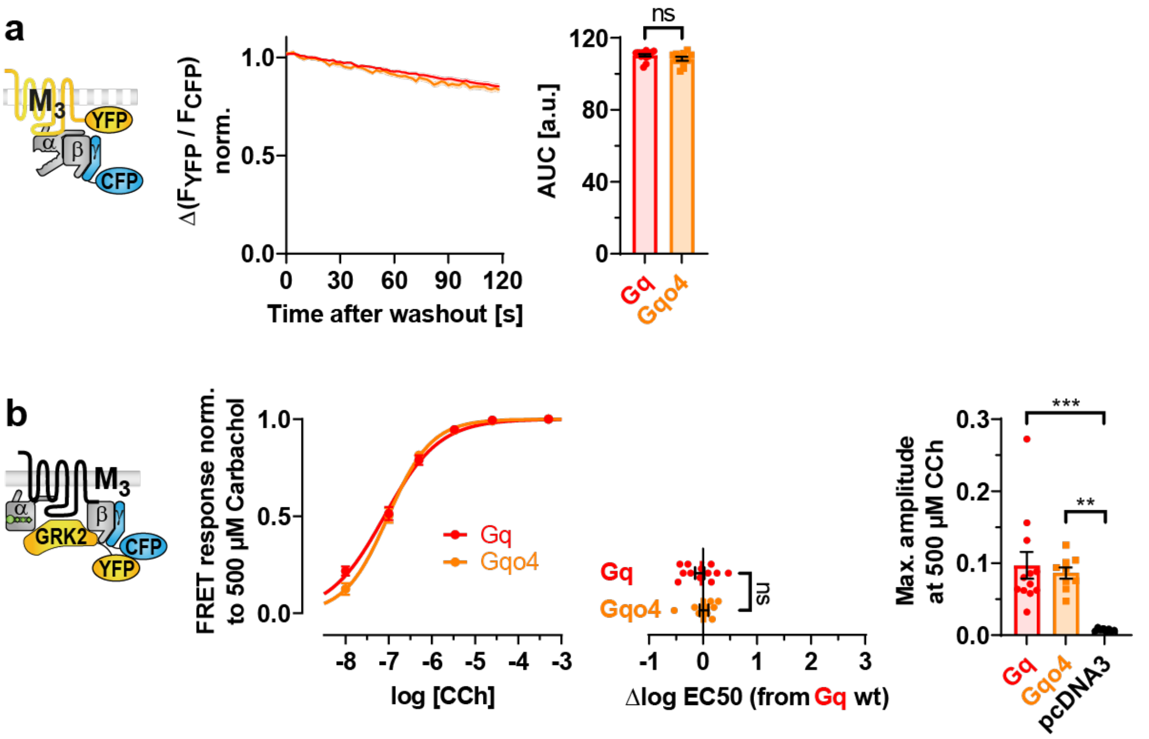


**Supplementary Fig. 11 Binding and activation measurements of Gqo4 chimeras with the $M_3$ receptor.** Gqo4 chimeras containing the α4/β6 loop of Gαo were investigated with the $M_3$R. **a** G protein binding experiments were performed in analogy to **Fig. 1b**. The dissociation kinetics of Gqo4 (orange; n=11) were similar to Gq (red; n=14). Data points are represented as means ± SEM from three independent experiments. **b** Indirect G protein activation measurements were performed in analogy to **Fig. 2a,b**. There was no detectable difference in the EC50 between Gqo4 (orange) and Gq (red). Data points are represented as means ± SEM from two independent experiments. (Gq; n=12, Gqo4; n=9; pcDNA3; n=8). Statistical analyses were performed using one-tailed t-tests for comparison of AUC and EC50 values or a one-way ANOVA with Dunnett's posttest to compare against pcDNA3 for the maximum amplitudes (**a-b**, ***$P < 0.001$, **$P < 0.01$, ns if $P \geq 0.05$).

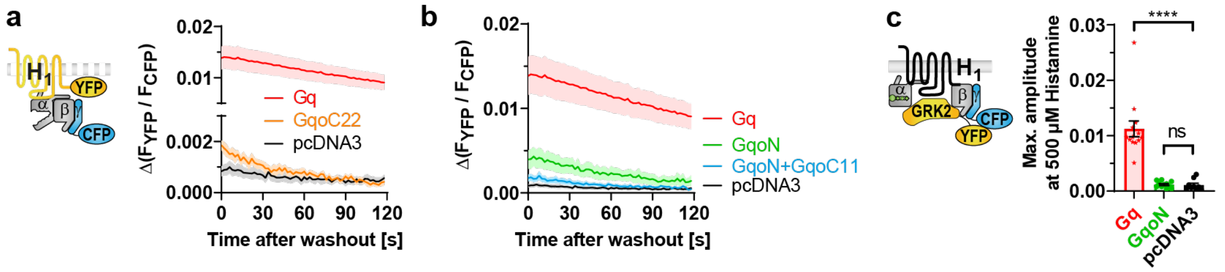


**Supplementary Fig. 12 Gα chimeras that cannot be bound or activated by the $H_1$-receptor. a-b** G protein binding experiments were performed in analogy to **Fig. 5a-e**. Averaged traces of absolute amplitudes for Gq (red; n=8), GqoC22 (orange; n=8), GqoN (green; n=6), GqoN+C11 (blue; n=9) and pcDNA3 (black; n=7) binding to $H_1$R illustrate that there were no clear signals of GqoC22 (**a**) and GqoN or GqoN+C11 (**b**) over endogenous G proteins (pcDNA3 as empty vector transfected instead of Gα). Data points are represented as means ± SEM from two independent experiments. **c** Indirect G protein activation measurements were performed in analogy to **Fig. 5f-j**. Maximum amplitudes of Gq (red; n=13), GqoN (green; n=11) and pcDNA3 (black; n=9) showed no specific activation of GqoN compared to endogenous proteins (pcDNA3). Data points are represented as means ± SEM from three independent experiments. Statistical analyses were performed using a one-way ANOVA followed by Dunnett's posttest to compare against pcDNA3 for the maximum amplitudes (****$P < 0.0001$, ns if $P \geq 0.05$).

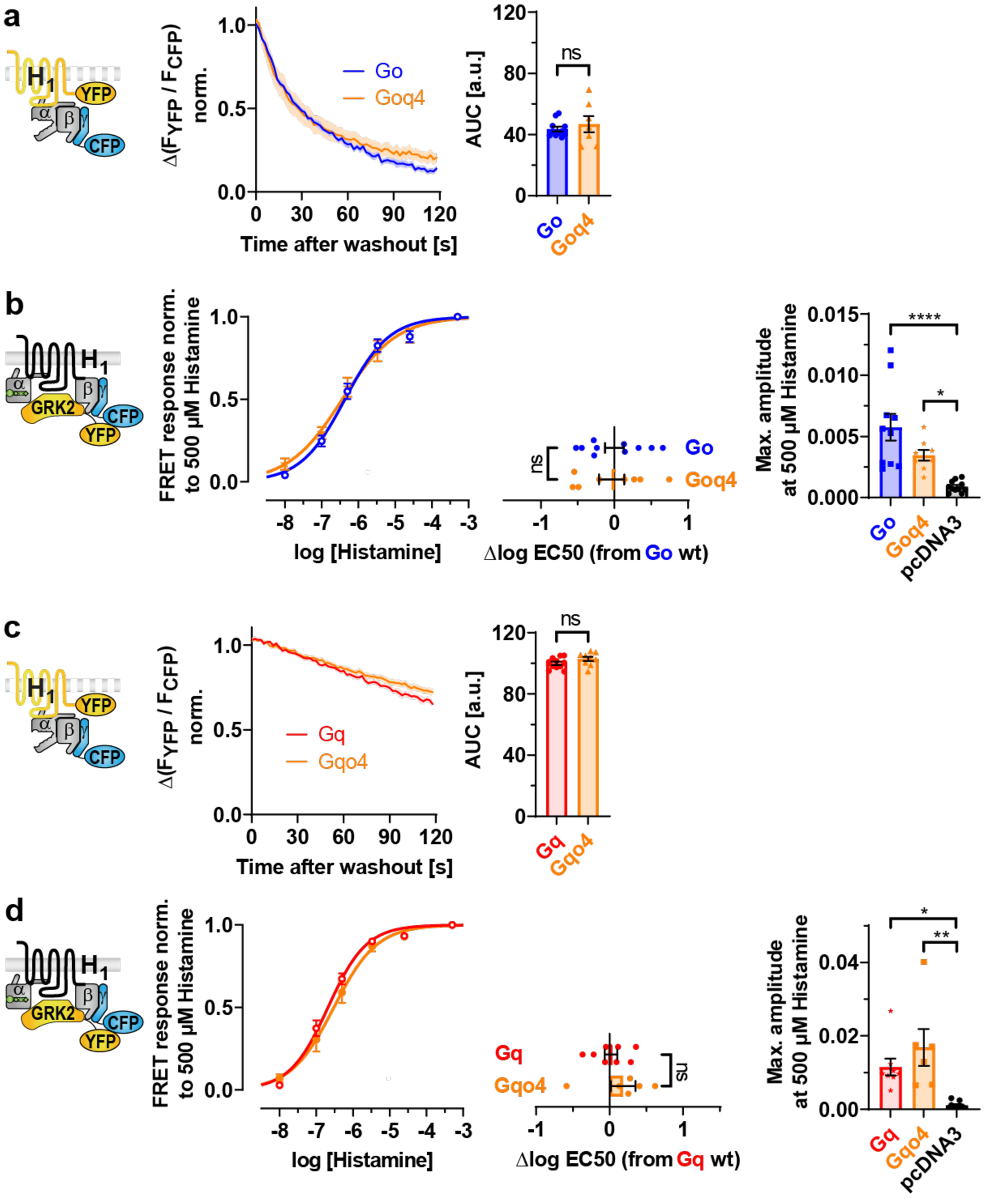


**Supplementary Fig. 13 Binding and activation measurements of Goq4 and Gqo4 chimeras with the $H_1$ receptor.** Goq4 and Gqo4 chimeras containing the α4/β6 loop of Gαo were investigated with the $H_1$R. **a** G protein binding experiments were performed in analogy to **Fig. 5a-e**. The dissociation kinetics of Goq4 (orange; n=7) from $H_1$R were similar to Go (blue; n=11). **b** Indirect G protein activation measurements were performed in analogy to **Fig. 5f-j**. There was no detectable difference in the EC50 between Goq4 (orange) and Go (blue). (Go; n=10, Goq4; n=8; pcDNA3; n=11). **c** In analogy to **a** the dissociation kinetics of Gqo4 (orange; n=9) from $H_1$R were similar to Gq (red; n=11). **d** In analogy to **b** there was no detectable difference in the EC50 between Gqo4 (orange) and Gq (red). (Gq; n=8, Gqo4; n=6; pcDNA3; n=9). All data points are represented as means ± SEM from at least two independent experiments. Statistical analyses were performed using one-tailed t-tests for comparison of AUC and EC50 values (**a-d**; ns if P ≥ 0.05) or a one-way ANOVA with Dunnett’s posttest to compare against pcDNA3 for the maximum amplitudes (**b,d**; ****P < 0.0001, **P < 0.01, *P < 0.05).

| Construct | Primer | DNA-Sequence (5'-3') |
|---|---|---|
| GoqC11 | vector fw | TCAAGGAGTACAATTTGGTCTAATCTAGAGGGCCCTATTCTATAGTGTCAC |
| | vector rv | GACCAAATTGTACTCCTTGAGATTGAGCTGAAGGATGATGTCGGTGACGGCG |
| GoqC22-11 | vector fw | CTTCGTCTTTGCAGCCGTCAAGGACACCATCATTGCCAACAATCTCCGGG |
| | vector rv | TGACGGCTGCAAAGACGAAGCGGATATTATTCGTGTCTGTGGCACAAGT |
| GoqC22 | vector fw | GTACAATCTGGTCTGAGCGGCCGCTCTAGAG |
| | vector rv | AAGCGGATATTATTCGTGTCTGTGGCACAAG |
| | fragment fw | GACACGAATAATATCCGCTTCGTCTTTGCAGC |
| | fragment rv | CCGCTCAGACCAGATTGTACTCCTTCAGGTTCAG |
| GqoC11 | vector fw | CCAACAATCTCCGGGGTATAGGTTTGTACTAAGCGGCCGCTCGAG |
| | vector rv | TATACCCCGGAGATTGTTGGCAATGATGGTGTCCTTGACGGCTG |
| GqoC22-11 | vector fw | GGTATTCGACGCCGTCACCGACATCATCCTGCAGCTGAACCTGAAGG |
| | vector rv | CGGTGACGGCGTCGAATACCACCTGGATGTTCTCGGTATCTGTGGCG |
| GqoC22 | vector fw | GTATAGGTTTGTACTAAGCGGCCGCTCGAG |
| | vector rv | CACCTGGATGTTCTCGGTATCTGTGGCG |
| | fragment fw | CGAGAACATCCAGGTGGTATTCGACGCC |
| | fragment rv | CGCTTAGTACAAACCTATACCCCGGAGATTGT |
| GoqN | vector fw | GAGCTCAAATTACTCCTGCTGGGGGCT |
| | vector rv | GTAAGCAGTGGGTTCTCTAGTTAGCC |
| | fragment fw | AGAACCCACTGCTTACTGGCT |
| | fragment rv | GCAGGAGTAATTTGAGCTCCCGGCGGG |
| GoqN+C11 | | GoqN exchange in GoqC11 |
| GoqN+C22 | | GoqN exchange in GoqC22 |
| Goq2 | vector fw | TCGACTTACAAAGTGTCATTTTCAGGCTGTTTGACGTTGGG |
| | vector rv | AATGACACTTTGTAAGTCGAAGTGGGTTTCTACGATGCCAG |
| Goq2+C11 | | Goq2 exchange in GoqC11 |
| Goq2+C22 | | Goq2 exchange in GoqC22 |
| GqoN | vector fw | CAAAGACGTGAAGCTGCTGCTGCTGGG |
| | vector rv | GTAAGCAGTGGGTTCTCTAGTTAGCC |
| | fragment fw | AGAACCCACTGCTTACTGGCT |
| | fragment rv | CAGCTTCACGTCTTTGGCGGCG |
| GqoN+C11 | | GqoN exchange in GqoC11 |
| GqoN+C22 | | GqoN exchange in GqoC22 |
| Gqo2 | vector fw | TTACCTTCAAGAACCTCCACTTCAGAATGGTCGATGTAGGGGG |
| | vector rv | GTGGAGGTTCTTGAAGGTAAAGGGGTATTCGATGATCCCTGT |
| Gqo2+C11 | | Gqo2 exchange in GqoC11 |
| Gqo2+C22 | | Gqo2 exchange in GqoC22 |

**Supplementary Table 1 Oligonucleotide sequences**

# Supplementary Methods

## Analysis of Gα expression levels

Gα expression levels were analyzed by immunoblots to confirm expression of the GqoC22-11 and GqoC22 chimeras which did not bind to the $M_3R$ and $H_1R$. The transfection contained the following amounts of plasmid cDNA: 0.5 µg of $M_3R$-mCit, 1.5 µg of the investigated Gα subunit, 0.5 µg $G\beta_1$ and 0.2 µg mTurq2-$G\gamma_2$. SDS-polyacrylamide gels (10%) were loaded with 30 µg protein (concentration was determined by a BCA protein assay) and after western blotting immunodetection was performed by the anti-Gαq antibody (Santa Cruz Biotechnology, Gαq (E-17), catalog#: sc-393, Lot#: 10214, rabbit polyclonal IgG, dilution 1:200 in 5% milk blocking buffer). The epitope recognition site within the N-terminus of Gαq enabled the detection of Gq-based chimeras containing the C-terminal helix of Gαo. The anti-actin antibody (MP Biomedicals, actin antibody (clone C4), catalog#: 691001, LOT#: Q2623, mouse monoclonal IgG1, dilution 1:100000 in 5% milk blocking buffer) was used as a loading control after membrane stripping. Anti-rabbit IgG and anti-mouse IgG HRP-linked antibodies (Cell Signaling Technology #7074, #7076) were used as secondary antibodies.

## Direct G protein activation assay

As a direct readout of G protein activation, FRET decreases can be measured upon dissociation of a YFP-labeled Gα and mTurquoise2-labeled Gβγ subunit if the receptor activates the G protein. The transfection contained 0.5 µg $M_3R$, 1.5 µg of Gα-YFP, 0.5 µg $G\beta_1$, 0.2 µg mTurq2-$G\gamma_2$. The measurement and data processing were performed in analogy to the previously described activation assay (see G protein activation assay: GRK2 recruitment by Gβγ subunit).

## Pertussis toxin pretreatment

Pertussis toxin was obtained as a glycerol solution (0.2 mg $mL^{-1}$). Before the experiments, cells were pretreated overnight with a concentration of 50 ng $mL^{-1}$ pertussis toxin in order to block PTX sensitive Gi/o proteins.
