# Supplementary material for: Structures in G proteins important for subtype selective receptor binding and subsequent activation
Source: Commun Biol. 2021 May 27;4:635. doi: 10.1038/s42003-021-02143-9 (PMC8160216; doi:10.1038/s42003-021-02143-9)
Supplement: Supplementary file 3 — Description of Additional Supplementary Files [file 42003_2021_2143_MOESM3_ESM.pdf]

Description of Additional Supplementary Files

File name: Supplementary Data 1

Description: Raw data for all main figures.
