# Supplementary material for: Structures in G proteins important for subtype selective receptor binding and subsequent activation
Source: Commun Biol. 2021 May 27;4:635. doi: 10.1038/s42003-021-02143-9 (PMC8160216; doi:10.1038/s42003-021-02143-9)
Supplement: Supplementary file 5 — Reporting Summary [file 42003_2021_2143_MOESM5_ESM.pdf]

nature research

Corresponding author(s): Moritz Bünemann

Last updated by author(s): Apr 14, 2021

Reporting Summary

Nature Research wishes to improve the reproducibility of the work that we publish. This form provides structure for consistency and transparency in reporting. For further information on Nature Research policies, see our Editorial Policies and the Editorial Policy Checklist.

Statistics

For all statistical analyses, confirm that the following items are present in the figure legend, table legend, main text, or Methods section.

n/a	Confirmed	
[ ]	[x]	The exact sample size (*n*) for each experimental group/condition, given as a discrete number and unit of measurement
[ ]	[x]	A statement on whether measurements were taken from distinct samples or whether the same sample was measured repeatedly
[ ]	[x]	The statistical test(s) used AND whether they are one- or two-sided<br>*Only common tests should be described solely by name; describe more complex techniques in the Methods section.*
[x]	[ ]	A description of all covariates tested
[x]	[ ]	A description of any assumptions or corrections, such as tests of normality and adjustment for multiple comparisons
[ ]	[x]	A full description of the statistical parameters including central tendency (e.g. means) or other basic estimates (e.g. regression coefficient) AND variation (e.g. standard deviation) or associated estimates of uncertainty (e.g. confidence intervals)
[x]	[ ]	For null hypothesis testing, the test statistic (e.g. *F*, *t*, *r*) with confidence intervals, effect sizes, degrees of freedom and *P* value noted<br>*Give P values as exact values whenever suitable.*
[x]	[ ]	For Bayesian analysis, information on the choice of priors and Markov chain Monte Carlo settings
[x]	[ ]	For hierarchical and complex designs, identification of the appropriate level for tests and full reporting of outcomes
[x]	[ ]	Estimates of effect sizes (e.g. Cohen's *d*, Pearson's *r*), indicating how they were calculated

Our web collection on statistics for biologists contains articles on many of the points above.

Software and code

Policy information about availability of computer code

Data collection	Data from the microscope was collected using Visiview 3 (Visitron Systems)
Data analysis	Data was analyzed using Microsoft Excel 2019, OriginPro 2018 and GraphPad Prism 8 as indicated in Methods section.

For manuscripts utilizing custom algorithms or software that are central to the research but not yet described in published literature, software must be made available to editors and reviewers. We strongly encourage code deposition in a community repository (e.g. GitHub). See the Nature Research guidelines for submitting code & software for further information.

Data

Policy information about availability of data

All manuscripts must include a data availability statement. This statement should provide the following information, where applicable:

- Accession codes, unique identifiers, or web links for publicly available datasets
- A list of figures that have associated raw data
- A description of any restrictions on data availability

The datasets generated and analysed during the current study are available from the corresponding author on reasonable request.

Field-specific reporting

Life sciences study design

All studies must disclose on these points even when the disclosure is negative.

Sample size	Sample sizes (n) for each experimental group are indicated in the Fig. legends.
Data exclusions	Data were only excluded if technical problems with the perfusion system occurred.
Replication	At least three independent experiments were performed for each experimental group
Randomization	Randomization was not relevant to these experiments.
Blinding	Blinding was not relevant to these experiments.

Reporting for specific materials, systems and methods

We require information from authors about some types of materials, experimental systems and methods used in many studies. Here, indicate whether each material, system or method listed is relevant to your study. If you are not sure if a list item applies to your research, read the appropriate section before selecting a response.

Materials & experimental systems

n/a	Involved in the study	
☐	☒	Antibodies
☐	☒	Eukaryotic cell lines
☒	☐	Palaeontology and archaeology
☒	☐	Animals and other organisms
☒	☐	Human research participants
☒	☐	Clinical data
☒	☐	Dual use research of concern

Methods

n/a	Involved in the study	
☒	☐	ChIP-seq
☒	☐	Flow cytometry
☒	☐	MRI-based neuroimaging

Antibodies

Antibodies used	Santa Cruz Biotechnology, anti-Gαq (E-17), catalog#: sc-393, Lot#: 10214, rabbit polyclonal IgG<br>MP Biomedicals, anti-actin (clone C4), catalog#: 691001, LOT#: Q2623, mouse monoclonal IgG1<br>Cell Signaling Technology, anti-rabbit IgG, HRP-linked Antibody (#7074), Lot#: 25<br>Cell Signaling Technology, anti-mouse IgG, HRP-linked Antibody (#7076), Lot#: 31
Validation	The epitope is recognized within the N-terminus of Gαq allowing to detect Gq-based chimeras containing the non-cognate C-terminal helix of Gαo

Eukaryotic cell lines

Policy information about cell lines

Cell line source(s)	Gift from the Lohse laboratory, University of Wuerzburg, likely from ATCC
Authentication	The HEK tsA201 cell line used in the study was not authenticated, since no endogenous signaling events were investigated
Mycoplasma contamination	All cell lines were regularly tested negative for myocoplamsa contamination.
Commonly misidentified lines (See ICLAC register)	Not applicable, since tsA201 cells are not on the list.
